# An Unusual Cause of Pancreatitis: Eclampsia

**DOI:** 10.7759/cureus.71342

**Published:** 2024-10-12

**Authors:** Govind Shiddapur, Sonali Agarwal, Asmita Samal, Yagnam Sai Hareeswar

**Affiliations:** 1 Department of General Medicine, Dr. D. Y. Patil Medical College, Hospital and Research Centre, Dr. D. Y. Patil Vidyapeeth (Deemed to be University), Pune, IND

**Keywords:** acute pancreatitis, microvascular thrombi, post partum pancreatitis, preeclampsia-eclampsia, pregnancy induced hypertension (pih)

## Abstract

Pancreatitis in pregnancy is not a common entity and has only been described with dysfunction of the biliary tract. Pregnancy in itself has not been described as a cause of pancreatitis, as is evidenced by the normal serum amylase and lipase values during the course of normal gestational periods. Pregnancy is known to be associated with hepatic dysfunction in the second or third trimesters, which can sometimes involve the pancreas but has not been documented to cause isolated involvement of the pancreas.

This gives us reason to believe that pregnancy-induced hypertension complicated with eclampsia is a rare but sinister and important differential in the peripartum period. Here is a case of a 25-year-old primigravida who presented to the hospital with eclampsia and developed pancreatitis on her second postoperative day in the hospital with no antecedent history of biliary disease, dyslipidemia, or alcohol consumption.

## Introduction

Acute pancreatitis is a rare complication of pregnancy [1]. About two of every three cases is biliary in origin, making gallstone pancreatitis the most common cause. Causes such as consumption of alcohol and hypertriglyceridemia may also be considered.

Pathologically, pancreatitis can have varied presentations [2]. It may range from interstitial pancreatitis to necrotizing pancreatitis. When the blood supply to the pancreas is maintained, it is labeled interstitial pancreatitis. This is generally easier to treat as it has a self-limiting course. When the blood supply is compromised, it leads to necrotizing pancreatitis. The release of enzymes such as pro-elastase, chymotrypsinogen, and trypsinogen causes widespread proteolysis. On the other hand, enzymes such as phospholipase A2 cause lipolysis [2]. Both of these processes are activated in the acinar cells, leading to injury of these cell compartments. Factors such as ischemia, infections (predominantly viral), toxin release (both endogenous and exogenous), release of lysosomal calcium, and sometimes even blunt trauma can cause catastrophic activation of trypsinogen to trypsin. Trypsin then activates other destructive proteolytic and lipolytic enzymes, which cause extensive damage to the cell structure, jeopardizing pancreatic function. Autodigestion can occur due to this spontaneous activation of trypsin [2].

Pregnancies complicated by pancreatitis often require multidisciplinary intensive care to prevent complications such as necrotizing or fulminant pancreatitis.

Here, we present a rare case of eclampsia leading to pancreatitis in a young primigravida patient.

## Case presentation

A twenty-five-year-old primigravida presented to the emergency department at thirty weeks of gestation with a history of two episodes of generalized tonic-clonic seizures in the past eight hours. Her antenatal period was uneventful, and she had no known medical co-morbidities. An emergency lower segment cesarean section was performed, and the pregnancy was terminated immediately. Both preoperatively and intraoperatively, the patient's blood pressure was 160/100 mm of Hg. Intravenous beta blockers were used initially, and the patient was initiated on oral calcium channel blockers. The patient was then shifted to the intensive care unit. On examination on days zero and one postoperatively, the patient was conscious, febrile, and in tachycardia. Her pulse rate was 122 beats per minute, and she had high blood pressure (170/110 mm of Hg). She was started on anti-hypertensives to control blood pressure, and oral feeds were planned to be initiated on day two of the postoperative period.

Consequently, the patient developed abdominal distension on day two with an increase in abdominal girth from 79 to 83 cm. The patient complained of loss of appetite, mild epigastric pain, and two episodes of vomiting that morning. On urgent sonography imaging of the abdomen and pelvis, moderate ascites were noted with free fluid in the perihepatic, Morrison’s pouch, right perinephric, perisplenic, inter-bowel, and pelvis regions. An abdominal paracentesis revealed transudative ascitic fluid (high serum-ascites albumin gradient (SAAG) ratio), turbid appearance, normal leukocyte count, and adenosine deaminase within normal limits. Ascitic fluid was sent for further examination, and it showed no growth in culture. The examination was only significant for elevated levels of ascitic amylase (3324 U/L) and lipase (12859 U/L).

On suspicion of acute pancreatitis, serum amylase, and lipase levels were sent and found to be elevated (serum amylase = 1190 U/L and serum lipase = 2319 U/L). A summary of all relevant laboratory investigations is provided in Table 1. Oral intake was restricted and the patient was managed with intravenous fluids.

**Table 1:** Laboratory values of the patient during her 18-day stay in the hospital TLC: total leukocyte count; SGOT: serum glutamic oxaloacetic transaminase; SGPT: serum glutamic pyruvic transaminase

Lab parameter	Day 1	Day 4	Day 10	Day 16	Reference range
Hemoglobin (g/dl)	9.3	9.3	10.20	9.10	13.2 - 16.6 g/dl
TLC	10500	16100	17800	8800	4000 – 100000/µL
Platelets	306000	105000	153000	221000	150000 – 410000/µL
SGOT	40	72	33	29	8 – 48 U/L
SGPT	26	35	31	27	7 – 55 U/L
Serum proteins	5.6	4.40	4.30	4.80	6.4 to 8.3 g/dl
Albumin	2.3	2.20	2.10	2.20	3.5 to 5.2 g/dl
Creatinine	1.07	0.98	0.80	0.57	0.6 to 1.2 mg/dl
Urea	21	32	18	18	17 to 49 mg/dl
Serum amylase	-	1190	572	114	28 to 100 U/L
Serum lipase	-	2319	1668	282	8 to 78 U/L

**Figure 1:**
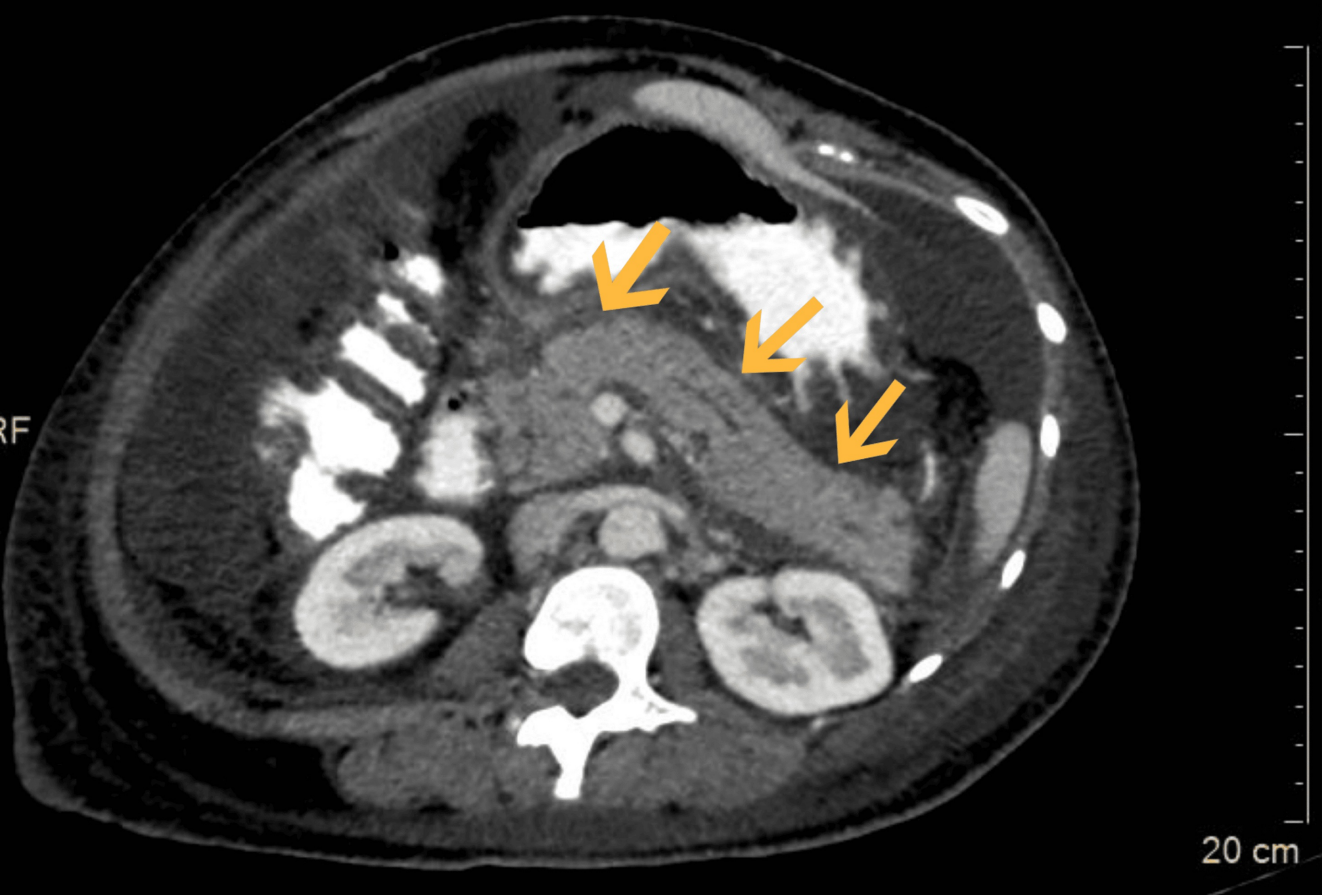
Axial sections of contrast-enhanced computed tomography of the abdomen and pelvis Contrast-enhanced computed tomography abdomen axial cut with oral contrast seen in the portal phase showing a bulky, uniformly, non-enhancing pancreas as indicated by the arrow marks.

**Figure 2:**
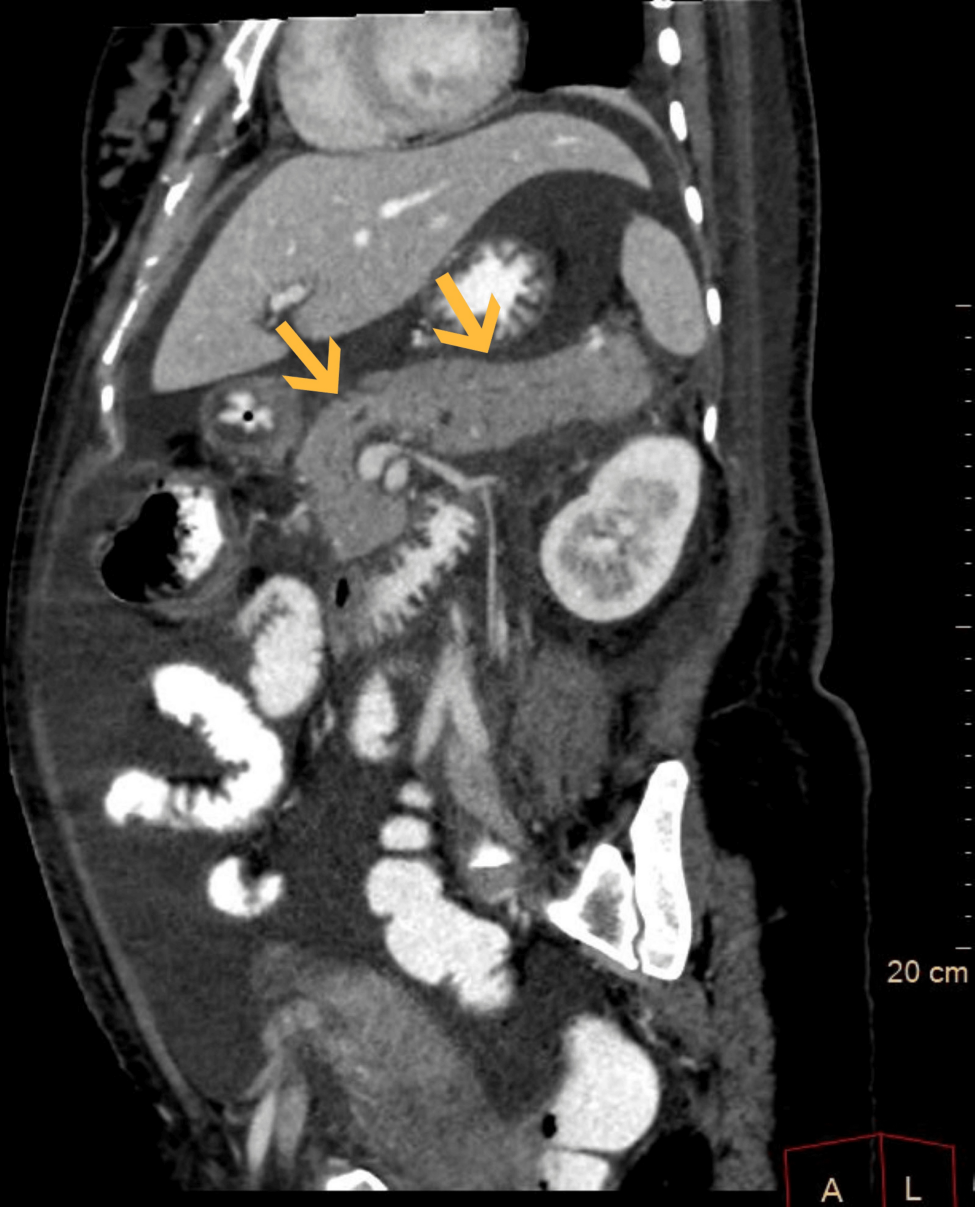
Sagittal sections of the contrast-enhanced computed tomography of the abdomen and pelvis The arrow marks indicate a non-enhancing pancreas in sagittal sections of the contrast-enhanced computed tomography of the abdomen and pelvis.

On contrast-enhanced computed tomography of the abdomen and pelvis done on day two, the pancreas appeared mildly bulky, and surrounding peripancreatic fat stranding was noted (Figure 1). The portal phase of the post-contrast study showed overall reduced enhancement of the pancreas. These findings (Figure 2) were suggestive of acute pancreatitis with a CT severity index of four.

The patient did not give a history of alcohol consumption or substance abuse. On contrast-enhanced computed tomography (CECT), the gall bladder was distended and appeared normal. No obvious radiodense calculus or mass lesion was visualized. A fasting lipid profile was within normal limits, details of which are provided in Table 2. Autoimmune workup for the patient was negative as well.

**Table 2:** Fasting lipid profile done for etiological evaluation VLDL: very low-density lipoproteins; HDL: high-density lipoproteins; LDL: low-density lipoproteins

Fasting lipid profile	Values (mg/dl)
Cholesterol	69
Triglycerides	91
VLDL	18
LDL	35
HDL	16

The patient was continuously monitored in the ICU over the next eight days. She experienced increased abdominal pain and distension over this period due to the accumulation of free fluid in the abdomen, which was relieved by performing a therapeutic abdominal paracentesis. She was started on a liquid diet from day five and a soft diet from day seven, which she tolerated well. She was treated with human albumin 20% injections for hypoproteinemia. The patient was passing stools every day, and bowel sounds were present.

Over the next week, the patient showed significant improvement and was shifted to the ward. Her blood pressure was under control, and her anti-hypertensive requirement was reduced. She was given the full course of antibiotics necessary and encouraged to hydrate well.

On discharge, the patient was on a single anti-hypertensive. She was eating well and was able to feed her newborn as well. Abdominal distension had subsided significantly with only occasional gastritis complaints at three months follow-up.

## Discussion

Acute pancreatitis is commonly associated with cholelithiasis, hypertriglyceridemia, and alcohol [3]. Pancreatitis is classically described as having three phases. The initial phase of pancreatitis is indeed characterized by intrapancreatic digestive enzyme activation and acinar cell injury. This activation can lead to inflammation and damage within the pancreas [2]. The second phase of pancreatitis involves the activation, chemoattraction, and sequestration of leukocytes and macrophages in the pancreas [2]. This inflammatory response contributes to tissue damage and further progression of the disease. The third phase of pancreatitis is characterized by the impact of activated proteolytic enzymes and cytokines released by the inflamed pancreas on distant organs.

Pregnancy-induced hypertension occurs due to the failure of the normal endovascular invasion of cytotrophoblast into the spiral arteries beyond the decidua-myometrial junction. Due to this failure of the formation of vascular endothelium, there occurs an imbalance of different components of prostaglandins. There occurs a deficiency of vasodilator prostaglandin (PGI2), an increase in thromboxane A2 (TXA2), a potent vasoconstrictor in platelets, a deficiency of nitric oxide, a vasodilator, the production of endothelin-1, a potent vasoconstrictor, an increase in tumor necrosis factor-α, and an increase in interleukin-6. This leads to widespread endothelial dysfunction and vasospasm. The microvascular changes in pre-eclampsia eclampsia syndrome may contribute to microthrombi, intravascular coagulation, and vasculitis [4]. A possible explanation of eclampsia-induced pancreatitis is the involvement and dysfunction of pancreatic vessels [5]. The treatment of eclampsia-induced pancreatitis includes aggressive fluid management [6], appropriate antibiotics, and adequate anti-hypertensive measures.

## Conclusions

Pancreatitis related to pregnancy is an entity that requires further evaluation and scrutiny. The usual etiology of pancreatitis does not seem to be the only cause for concern in pregnant females. Eclampsia or pre-eclampsia-induced hypertension causing pancreatic microvascular compromise is a cause that has rarely been considered in the past. Eclampsia in itself is a high-risk emergency that has now emerged with yet another serious complication: acute pancreatitis. Postpartum females with a history of pregnancy-induced hypertension presenting with abdominal distension and pain must be evaluated for pancreatitis. Simple blood tests such as serum levels of amylase and lipase that are readily available in most labs must be used in addition to imaging to confirm the diagnosis. Iatrogenic injury post-surgery must be ruled out as well in such a setting.

The management of pancreatitis depends on the grade and severity of the disease and follows the usual guidelines as in nonpregnant females. Adequate fluid resuscitation and empirical antibiotic coverage form the cornerstone of management. Early diagnosis and initiation of therapy prove to be the most vital aspect of treatment. Hence, a consideration of this complication in eclampsia-pre-eclampsia patients in their postnatal period is important.
